# NGS-Trex: Next Generation Sequencing Transcriptome profile explorer

**DOI:** 10.1186/1471-2105-14-S7-S10

**Published:** 2013-04-22

**Authors:** Ilenia Boria, Lara Boatti, Graziano Pesole, Flavio Mignone

**Affiliations:** 1 Dipartimento di Chimica, Università degli Studi di Milano, Milano, Italy; 2 Dipartimento di Scienze e Innovazione Tecnologica, Università del Piemonte Orientale, Alessandria, Italy; 3 Dipartimento di Bioscienze, Biotecnologie e Scienze Farmacologiche, Università degli Studi di Bari "A. Moro", Bari, Italy; 4 Istituto di Biomembrane e Bioenergetica, Consiglio Nazionale delle Ricerche, Bari, Italy

## Abstract

**Background:**

Next-Generation Sequencing (NGS) technology has exceptionally increased the ability to sequence DNA in a massively parallel and cost-effective manner. Nevertheless, NGS data analysis requires bioinformatics skills and computational resources well beyond the possibilities of many "wet biology" laboratories. Moreover, most of projects only require few sequencing cycles and standard tools or workflows to carry out suitable analyses for the identification and annotation of genes, transcripts and splice variants found in the biological samples under investigation. These projects can take benefits from the availability of easy to use systems to automatically analyse sequences and to mine data without the preventive need of strong bioinformatics background and hardware infrastructure.

**Results:**

To address this issue we developed an automatic system targeted to the analysis of NGS data obtained from large-scale transcriptome studies. This system, we named NGS-Trex (NGS Transcriptome profile explorer) is available through a simple web interface http://www.ngs-trex.org and allows the user to upload raw sequences and easily obtain an accurate characterization of the transcriptome profile after the setting of few parameters required to tune the analysis procedure. The system is also able to assess differential expression at both gene and transcript level (i.e. splicing isoforms) by comparing the expression profile of different samples.

By using simple query forms the user can obtain list of genes, transcripts, splice sites ranked and filtered according to several criteria. Data can be viewed as tables, text files or through a simple genome browser which helps the visual inspection of the data.

**Conclusions:**

NGS-Trex is a simple tool for RNA-Seq data analysis mainly targeted to "wet biology" researchers with limited bioinformatics skills. It offers simple data mining tools to explore transcriptome profiles of samples investigated taking advantage of NGS technologies.

## Background

Despite Next-Generation Sequencing (NGS) technologies are becoming increasingly accessible and cost effective and a large number of analysis tools are regularly made available to the research community, the throughput level reached and the complexity of the analysis still poses serious problems related to the management and the interpretation of data.

While the conceptual analysis pipeline to deal with NGS data is relatively easy to be drawn, the actual implementation can be challenging. Indeed, the simple mapping of sequences onto a reference genome requires adequate computational power and properly set-up systems. Things gets even more complicated when it comes to compare data with annotated features because of the need of up-to-date databases and automated analysis procedures.

Even if many tools are available to perform those tasks (for a recent review and comparison see [1]) - also allowing the usage of remote servers thus reducing the hardware and software requirements - they still require some skills to be installed or their usage needs suitable training.

The efforts needed to set up the infrastructure and to acquire the required skills are beyond the possibility of many laboratories and are often not justified for projects where only few deep sequencing cycles are needed to address specific biological questions.

To fill this gap we developed an automatic system targeted to the analysis of Next Generation Sequencing data obtained from large-scale transcriptome studies. This system, we named NGS-Trex (NGS Transcriptome profile explorer) is available through a simple web interface http://www.ngs-trex.org and besides requiring a simple user interaction it offers an accurate characterization of the transcriptome profile of the samples under investigation. The system is also able to assess differential expression of genes at both gene and transcript level (i.e. splicing isoforms) by comparing the expression profile of different datasets.

Among the different tools available the only one that can be directly compared to NGS-Trex is the recently published GeneProf system [1]. GeneProf http://www.geneprof.org/GeneProf/ and NGS-Trex share the same philosophy being both web-based and intended to offer an "easy to use" tool for RNA-Seq data analysis. Also the well-established Galaxy tool [2] can be used to analyse RNA-Seq data. However Galaxy is a more general purpose framework and it requires a strong user interaction at each stage of the workflow resulting in a relatively complex tool that requires a steep learning curve.

## Results

### Analysis overview

To complete the overall procedure the user has to perform three simple steps: 1) create a "project" and upload the sequences; 2) tune the analysis parameters; 3) mine data for relevant information.

**Figure 1:**
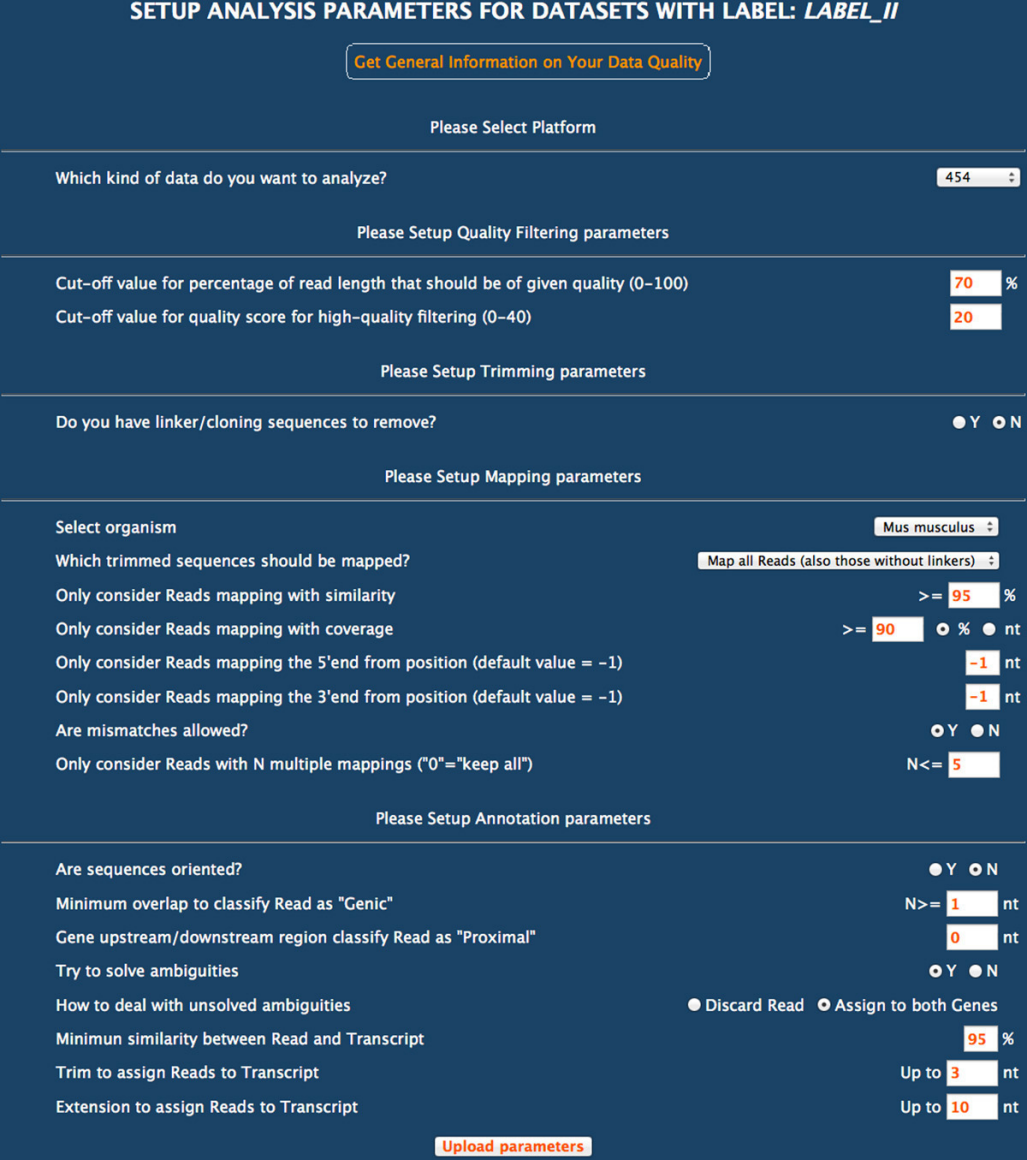
**Setup analysis form**. Snapshot of the form for the setup of all the parameters needed to perform the three steps of the analysis (pre-processing of sequences, mapping onto the reference genome, comparison to the available annotation). All fields are pre-compiled with default values.

All datasets within the same project - besides being processed with the standard analysis procedure - are automatically compared each other to identify differentially expressed genes or differentially represented splice sites (i.e. introns). Data can be analysed with the default options but it is also possible to tune the procedure by setting few parameters through a very simple form as shown in Figure 1. This allows the user to control sequences pre-processing, read mapping criteria against the reference genome and annotation strategy as described below.

### Data analysis

The basic analysis workflow implemented in our tool follows a common schema for RNA-Seq data. It is basically articulated into 4 steps: 1) sequence filtering to discard low quality reads, to remove cloning linkers and to identify, whenever possible, sequence strandness; 2) mapping of reads onto the reference genome; 3) comparison of mapped reads to annotated features; 4) identification of "interesting" features (such as highly expressed genes, un-annotated splicing events, significant differences in expression levels).

**Figure 2:**
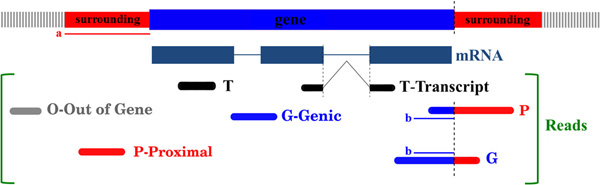
**Read to gene assignment confidence**. Reads are assigned to genes with three different levels. Proximal (P) reads are those mapping close to gene boundaries, Genic (G) reads overlap gene coordinates, Transcript (T) reads show ungapped matches with RefSeq transcripts annotated as part of the gene model, Extragenic reads (O) represent reads falling outside gene coordinates beyond the defined "surrounding" region. Both the extension of the "surrounding region" (thin red line labeled as "a" in figure) and the minimum overlap with gene mapping region to classify reads as "G" (thin blue line "b") can be defined by the user.

Through the filtering step it is possible to trim low quality sequences and to remove adapters that may be part of the reads. If different adapters were used for 5' and 3' ends, it is also possible to orient reads improving the subsequent annotation procedure. Reads are then mapped onto the reference genome using two different algorithms depending on the sequencing technology: gmap [3] for longer sequences (such as Roche 454 reads) and TopHat [4] for shorter reads (i.e. Illumina or SOLiD). Mapped sequences that satisfy a filtering threshold defined by minimum overlap, minimum similarity and maximum number of multiple matches over the reference genome are selected for the annotation process. The annotation process is a multi-step procedure that ends with the assignment of reads to gene-specific clusters. We assign reads to genes with three degrees of confidence: reads mapping to a region flanking the gene (within a user-defined range) are tagged as proximal "P", reads overlapping gene coordinates are tagged as genic "G", reads showing contiguous mapping to RefSeq transcripts and not extending them over a user-defined threshold are tagged as "Transcript reads" "T". Figure 2 shows a graphical representation of the assignment process.

The annotation process is very critical and several factors may affect the quality of the annotation. The main problem is related to ambiguities in the correct assignment of a read to a gene [5]. Two distinct classes of ambiguities can be identified: we define class I ambiguities those generated by overlapping genes and class II ambiguities those caused by reads which are not uniquely mapped on the genome (mainly due to paralogues genes, pseudogenes or gene clusters).

Several analysis workflows simply bypass class II ambiguities by discarding sequences not uniquely mapped. The main problem with this approach is the introduction of an experimental bias with the alteration of reads count of gene families. Our system attempts to solve ambiguities using information about relative orientation of genes, about confidence of the assignment of the read to ambiguous genes and relying on strand information obtained from spliced reads. Indeed donor and acceptor sites of spliced reads can be used to guess the correct strand of reads when they are not oriented.

If reads are oriented (or spliced) and overlapping genes are on opposite strands, ambiguities can be easily solved and the read is assigned to the gene on the same strand; conversely if reads are not oriented and/or genes are on the same strand the correct assignment of a read to the right gene is more challenging. However, a reasonable solution to this issue is the assignment of the read to the competing genes with different level of confidence (the order of assignment of read is "T" > "G" > "P"). Whenever an ambiguity cannot be solved the user can choose either to discard the read or to assign the same read to both genes.

### Finding "interesting features"

In addition to read assignment to genes and transcripts NGS-Trex identifies un-annotated splice sites. Putative new splice sites are classified according to their donor/acceptor sites and to the number of supporting reads. However they are not assembled into transcript models.

Finally, whenever two or more datasets are available for the same project, the system automatically identifies genes and introns that show a differential expression pattern. As pointed out in [6] a standard procedure for the identification of differentially expressed (DE) genes is not yet available. Moreover current implementation of DE genes calculation in NGS-Trex treats each pair separately (biological replicates - if available - are not taken into account). In this context Fisher's exact test appears adequate to reliably explore putative DE genes [7] and was implemented in our tool.

### Data mining tools

Upon completion of the analysis process statistical summaries of data are provided together with several data mining tools.

Statistics show summary information about parameters used for the analysis, lengths distribution charts for both submitted and processed reads and flowchart of reads classified during the mapping and annotation processes. Finally, an overview of specific features like the number of novel introns, differentially expressed genes or introns is shown.

**Figure 3:**
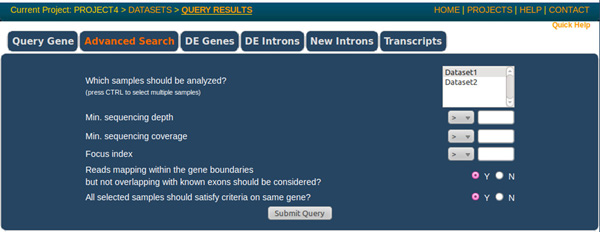
**Data-mining forms**. Upon the completion of the analysis it is possible to mine information about single genes (Query Gene) or obtain lists of most represented genes in the samples (Advanced Search). DE Genes and DE Introns panels allow a simple exploration of putative differentially expressed genes or splicing events. It is also possible to identify un-annotated introns (New Introns) and to investigate reads assigned to RefSeq mRNAs (Transcripts).

As depicted in Figure 3 it is possible to perform several queries on data by accessing very simple query forms. The "Query gene" panel allows a rapid search of a single gene using either the *entrez gene ID *or the *hugo *accession. The result is a brief summary showing the count of reads assigned to the gene and the count of both annotated and new introns identified by the analysis. Furthermore the differential expression pattern of the selected gene within the different datasets is provided. With the "Advanced search" panel it is possible to rank genes that satisfy several criteria. In particular three indexes are available: "coverage", the total number of sequences assigned to the gene; "depth", the maximum number of reads covering a specific genic position; "focusing index", the ratio between depth and coverage. If all or most of the reads map in a single position along the gene, the focusing index is high thus implying that the reads likely match a repetitive sequence. The lower is the focusing index, the more homogeneous is the distribution of aligned reads along the gene under investigation. When multiple datasets are queried it is possible to apply search criteria as intersection or union among datasets.

"DE genes" and "DE introns" panels can be used to highlight genes and introns that are significantly differentially expressed, i.e. up- or down-regulated between a reference and the others datasets.

Finally, with "New introns" query form it is possible to obtain a list of un-annotated splicing events and with "Transcripts" query panel it is possible to investigate the relation between reads and known transcripts by identifying the relative position of reads respect to CDS and UTRs regions.

Results are shown as tables and can be downloaded as tab-delimited text file to be easily imported into a spreadsheet program. A basic genome browser interface is also available to allow visual inspection of data and to simplify the download of reads assigned to a gene or mapped onto a genomic region.

### Benchmark

To benchmark our system we processed the sequences obtained by [8] with Titanium 454-Roche-platform. Sequences are available at the NCBI Sequence Read Archive (SRA, http://www.ncbi.nlm.nih.gov/Traces/sra) with accession SRA012436. The Authors carefully analysed data and performed experimental validation to confirm among other findings - splicing variants, enrichment in alternative splicing events and alterations in expression levels of several genes. Sequences were obtained from a pool of cDNA produced from two distinct cell lines HB4a and HB4aC5.2. A barcoding sequence was inserted to discriminate between the two datasets.

We firstly analysed the whole dataset mainly using our default values (we only tuned overlap and minimum similarity threshold for the mapping step to match the same values used in the paper: 70% and 96% respectively).

Table 1 summarizes the comparison between the results shown by Carraro et al. [8] and the results obtained with NGS-Trex analysis. It is important to underline that all data reported in the table are readily available from the web interface in the summary table or in the statistical report and show good correlation with data obtained by Carraro et al.

**Table 1:** Benchmark.

**Label given in Carraro et al**.	**Carraro et al**.	NGS-Trex	Corresponding label in NGS-Trex
Total reads	802214	802214	Total reads (1, 2)

Processed reads	731628	796352	Processed reads (2)

Partly aligning to Human Genome	80570	84593	Mapped low similarity (2)

Completely aligning to Human Genome	651058	691530	Mapped reads (1, 2)

Mapped at one genome position	614434	563190	Mapped with low frequency (2)

Mapped against Known Gene DB	597565	539492	Genic (2)

Mapped against RefSeq	476337	466524	Classified as "T" (2)

Represented mRNAs	17887	20500	Identified transcripts (2)

Represented genes	11366	13415	Identified genes (1, 2)

Summary of the comparison between the results shown by Carraro et al. [8] and the results obtained from NGS-Trex analysis. Data shown in the table are available from the summary table of the project in NGS-Trex website (1) or in the statistical report linked to the dataset (2). Differences can be mainly explained by different programs used, annotation releases and sources (UCSC genome browser data were used by Carraro, while NGS-Trex mainly relies on annotation data available from NCBI).

To identify DE genes we processed again the sequences using the barcoding information to discriminate between the two datasets. While NGS-Trex does not explicitly deal with pooled samples it is easy to obtain this feature creating as many datasets as the barcodes used and defining the proper barcode sequence as the linker in the filtering step of the procedure.

Comparison of fold changes and pValues of genes identified as differentially expressed by Carraro with those extracted by NGS-Trex web interface is available in Additional File 1. Fold changes are highly correlated between the two studies (rho = 0.775, p-Val < 2.2e-16) as depicted in Additional File 2 (kindly provided by one of the reviewers) reinforcing the reliability of the results obtained from our approach. Finally, we focused on new splicing events. Carraro and colleagues identified 2865 putative new splicing events classified as intron retention, alternative exon usage and alternative splice sites events (see Figure 4 in Carraro paper [8] or a description of the different classes). Those data are not directly comparable with our results because we use a different approach: while results shown in the paper deal with splicing from the "exon point of view", NGS-Trex treats splicing from the "intron point of view". So - as an example - we cannot explicitly identify a cassette-exon event but only the two distinct flanking introns. For this reason, considering that we do not identify intron retention events and that each exon-inclusion event is generated by 2 splice sites, the 2865 new splice variants suggested in the paper reflect - from NGS-Trex perspective - 1757 events. With our analysis we identified 1641 un-annotated introns (1253 with canonical Donor/Acceptor site), 683 of which confirmed by at least 2 reads (542 with canonical D/A site). All the experimentally validated splicing events have also been identified by our analysis (Table 2).

**Table 2:** Splicing variants.

Novel AS Events	**Carraro et al**.	Carraro et al. (*)	NGS-Trex (N > 1)	NGS-Trex (N > 2)
Total	2865	1757 (**)	1641	683

Canonical	na	na	1245	539

RT-PCR validation	18	36	36	

qRT-PCR	8	16	16	

As described more in detail in the text, NGS-Trex treats splicing events from the "intron point of view" while in Carraro work splicing are classified from the exon perspective. This implies that one cassette-exon event is identified by NGS-Trex as two splicing sites. To compare data we corrected data to take into account this difference. Column marked with (*) shows the corrected values. (**) 1757 events derive from 530 AS donor/acceptor site usage + 487 exon skipping + 562 exon inclusion + 89×2 exon inclusion (each exon inclusion needs 2 introns). See also Figure 4 in Carraro paper for details.

It is beyond the scope of this discussion to make a detailed comparison between the results obtained from the two methods. The differences can be explained by different parameters and software used. The goal of this comparison was simply to demonstrate that results obtained with our system with a very simple submission of sequences, without requiring a deep knowledge of underlying analysis procedures, are qualitatively comparable to those obtained from a manually curated analysis that involves the combination of custom procedures with the usage of external tools.

### Comparison with other tools

Several tools are already available for the analysis of NGS data (for a detailed comparison table see supplementary note in [1]). Most of them require complex installation procedures and have specific hardware requirements or need a strong user interaction to perform the analysis. The only available tool comparable to NGS-Trex is - to our knowledge - GeneProf [1]. Nonetheless, this tool seems more focused on the early stage of the analysis: it offers automatic tools to import sequences from SRA database and shows many plots about sequence quality, base composition and read alignment statistics. On the other hand we were not able to find information about splicing or about annotation at transcript level. Finally, we did not find any method to download specific sequences set (i.e. reads assigned to a specific gene). This is quite important from a "wet biologist" perspective as it allows to carefully inspect sequences to identify mutations or to design primers for experimental validation of observed data.

We do not exclude that it is possible to extract these information by using GeneProf as a more experienced user but to this respect NGS-Trex seems more user-friendly.

## Discussion

NGS-Trex is a user-friendly system to analyse RNA-Seq data. Indeed, to our knowledge any such web tool to visualize and download data mining results as well as specific subset of sequence reads (i.e. those assigned to a specific gene, supporting a splice site, etc.) is not currently available. These read subsets can be used for further analyses by using third part software. For example, the current NGS-Trex version does not allow the direct identification of SNPs, nonetheless, while is not possible to obtain a genome wide identification of SNPs, using the exploration and extraction tools currently provided it is very easy to download sequences assigned to a specific gene and - by using external resources - process only the relevant sequences to obtain the required result.

The reliability of results provided by NSG-Trex has been supported by the benchmark assessment taking into account that observed discrepancies may be explained as the result of different tools and parameters as well as by difference in the genome feature annotation considered. However, our benchmark assessment has shown that results obtained by our system are quite comparable to those obtained from a manually curated analysis that involves the combination of custom procedures with the usage of external tools.

## Methods

### Implementation

The analysis workflow has been implemented as a combination of custom perl and php scripts. Data are stored as a combination of flat files and relational databases using Mysql RDBMS. As part of the analysis several tools have been included in the pipeline, namely 1) NGS QC Toolkit [9] for the processing of fastq sequences, 2) gmap (v. 2011-10-04) and 3) TopHat (v. 2.0.6) for alignment step. All programs are used with default values.

Comparison with annotation is performed using a custom database which is mainly populated with several sources obtained from NCBI ftp site. Genes mapping information is derived from NCBI while RefSeq and Genbank sequences (assigned to genes as part of Unigene clusters) are mapped onto the reference genome by using gmap software. Sequences are updated every 6 months.

In NGS-Trex are currently available *Homo sapiens *(Hg18, Hg19), *Mus musculus *(mm9) and few bacteria. More genomes will be added also upon request. As previously described a critical step in the analysis procedure is the read assignment in the case of ambiguities. The user can define whether to discard ambiguously assigned reads or to assign those reads to all competing genes. Moreover it is possible to trigger a procedure to try to solve ambiguities. This procedure adopts several strategies: the first criterion is the confidence level of read assignment, being the order from higher to lower "T", "G", "P" (see text and Figure 2 for details). Whenever a read is assigned to more than one gene with the same confidence the procedure can still solve ambiguities for spliced reads if 1) the read supports an annotated splicing site, 2) the read is not oriented but the correct strand can be guessed using donor acceptor sites (in this case the read is treated as oriented and it is assigned to the gene on the same strand). If - after this procedure - it is still not possible to assign a read to a single gene it can be either assigned to all competing genes or discarded.

Differential expression (both at gene and splice site level) is evaluated applying Fisher's exact test. Read counts from different samples are normalized by the total number of reads mapped onto the genome.

Concerning input data, NGS-Trex can handle long reads (454-Roche) and short reads (Illumina), with fixed or different lengths. Files in fasta or fastq formats are accepted. Currently paired-end reads are not supported (although they can be analysed as single end). Multiplexed run can be processed.

## Competing interests

The authors declare that they have no competing interests.

## Authors' contributions

IB participated in the design of the study and worked at the implementation of the analysis workflow and of the website. LB carried out the validation benchmark. GP participated in the design of the study and helped to draft the manuscript. FM conceived the study, worked at the implementation of the analysis workflow and drafted the manuscript. All authors read and approved the final manuscript.

## Declarations

This article was supported by Ministero dell'Istruzione, dell'Università e della Ricerca: FIRB "Futuro in ricerca" code RBFR08PWXIF.

## Supplementary Material

Additional File 1 **Differentially expressed genes**. List of differentially expressed genes validated in Carraro et al. [8]. Reads count of data obtained from Carraro are expressed as reads per million (RPM) and when no reads were identified in the RNA-Seq from one of the cell lines "0" was replaced by "1". Reads counts obtained from NGS-Trex analysis are absolute (a scaling factor of 3.88 for Hb4a and 2.80 for C5.2 can be applied to transform counts into RPM). When no reads were identified from one of the cell lines the fold change is "nd". Click here for file

Additional File 2 **Correlation of fold change values**. The plot shows the correlation between Carraro and NGS-Trex fold changes for the 88 genes listed in Additional File 1. Click here for file
