# Supplementary material for: NGS-Trex: Next Generation Sequencing Transcriptome profile explorer
Source: BMC Bioinformatics. 2013 Apr 22;14(Suppl 7):S10. doi: 10.1186/1471-2105-14-S7-S10 (PMC3633008; doi:10.1186/1471-2105-14-S7-S10)
Supplement: Additional File 1 — Differentially expressed genes. List of differentially expressed genes validated in Carraro et al. [8]. Reads count of data obtained from Carraro are expressed as reads per million (RPM) and when no reads were identified in the RNA-Seq from one of the cell lines "0" was replaced by "1". Reads counts obtained from NGS-Trex analysis are absolute (a scaling factor of 3.88 for Hb4a and 2.80 for C5.2 can be applied to transform counts into RPM). When no reads were identified from one of the cell lines the fold change is "nd". [file 1471-2105-14-S7-S10-S1.pdf]

Supplementary Table 1

* count expressed as reads per million (rpm)
** when no reads were identified in RNA-seq from one of the cell lines ‘‘0’’ was replaced by ‘‘1’’
reads count in “NGS-Trex G+T” and “NGS-Trex T” panels are absolute and when no reads were identified in RNA-seq from one of the cell lines the fold change is “nd”

Gene Name	Carraro et al.				NGS-Trex G+T					NGS-Trex T				
	**C5.2 ***	**Hb4a ***	**Fold change ****	**Fc (qRT-PCR)**	**C5.2**	**Hb4a**	**Fold change**	**pValue**	**pValue adj**	**C5.2**	**Hb4a**	**Fold change**	**pValue**	**pValue adj**
ALDH2	14	0	14	41	20	0	nd	1.89E-05	2.28E-04	18	0	nd	5.61E-05	5.39E-04
ALDOA	1919	458	4	1	492	83	4	2.13E-46	3.92E-44	406	72	4.1	5.47E-37	8.84E-35
ANGPTL4	246	7	35	7	73	4	13	2.60E-13	1.02E-11	66	3	15.9	1.07E-12	3.93E-11
ANXA6	13	0	13	2	21	0	nd	1.10E-05	1.40E-04	18	0	nd	5.61E-05	5.39E-04
ATP5G3	685	14	49	2	254	8	23	4.99E-49	9.79E-47	205	8	18.5	3.54E-38	6.08E-36
ATP5L	568	55	10	2	178	13	10	5.99E-28	5.99E-26	168	12	10.1	1.14E-26	1.12E-24
C12orf44	146	14	10	2	33	2	11.9	1.83E-06	2.78E-05	29	2	10.5	1.27E-05	1.48E-04
CAPG	720	96	8	1	161	16	7	2.21E-22	1.74E-20	143	14	7.4	3.54E-20	2.56E-18
CDC20	392	89	4	1	100	18	4	4.04E-10	1.13E-08	82	16	3.7	5.07E-08	9.87E-07
COL3A1	28	0	28	493	44	0	nd	4.07E-11	1.28E-09	42	0	nd	1.21E-10	3.51E-09
COPE	170	27	6	1	104	8	9	1.40E-16	7.18E-15	102	6	12.3	9.22E-18	5.41E-16
COX11	20	0	20	2	26	4	5	7.61E-04	5.78E-03	17	2	6.1	3.39E-03	1.80E-02
COX4I1	562	14	40	2	181	9	15	5.63E-32	6.73E-30	148	7	15.3	1.01E-26	9.92E-25
COX8A	1293	294	4	1	350	70	4	9.11E-29	9.64E-27	275	48	4.1	6.39E-26	5.84E-24
CSDA	199	14	14	2	55	2	20	3.03E-11	9.70E-10	38	2	13.7	1.56E-07	2.80E-06
DST	35	212	-6	1	12	49	-6	9.95E-10	2.70E-08	11	43	-5.4	1.74E-08	3.72E-07
EEF1A1	4781	96	50	-1	2232	133	12	0.00E+00	0.00E+00	1857	123	10.9	1.26E-287	1.43E-283
EEF1B2	263	48	5	1	181	83	2	2.85E-04	2.48E-03	154	79	1.4	7.29E-03	3.35E-02
EIF4EBP1	199	41	5	1	46	10	3	1.15E-04	1.10E-03	45	8	4.1	2.61E-05	2.76E-04
ERBB2IP	18	137	-8	1	4	31	-11	1.31E-08	2.91E-07	4	31	-10.7	1.31E-08	2.86E-07
FAU	158	14	11	1	66	3	16	1.07E-12	3.98E-11	55	3	13.2	2.52E-10	7.13E-09
FBXL6	26	0	26	2	37	0	nd	1.83E-09	4.79E-08	29	0	nd	1.42E-07	2.55E-06
GALNT3	6	267	-45	-2	2	53	-37	5.14E-18	2.95E-16	2	45	-31.1	3.93E-15	1.85E-13
HMGA1	281	27	10	2	69	6	8	6.29E-11	1.92E-09	64	6	7.7	6.27E-10	1.67E-08
HMGB1	556	82	7	2	203	25	6	7.35E-25	6.32E-23	195	24	5.9	6.00E-24	4.82E-22
HMGB2	20	0	20	1	35	1	25	8.51E-08	1.65E-06	35	0	nd	5.43E-09	1.26E-07
HMGN2	374	7	53	1	158	18	6	1.54E-20	1.13E-18	139	13	7.7	4.63E-20	3.26E-18
HRAS	257	41	6	2	57	8	5	2.05E-07	3.78E-06	55	8	5.0	4.71E-07	7.66E-06
HSPA8	544	75	7	4	181	30	4	2.56E-18	1.52E-16	168	26	4.7	5.37E-18	3.19E-16
HSPE1	445	7	64	2	167	1	100	2.56E-38	3.88E-36	122	1	88.1	8.01E-28	8.40E-26
IL6ST	6	226	-38	1	884	48	13	7.96E-147	7.31E-144	1	37	-51.2	2.45E-13	9.58E-12
JUP	23	68	-3	1	7	13	-3	3.20E-02	1.16E-01	7	13	-2.6	3.20E-02	1.05E-01
KRT15	1129	164	7	3	274	34	6	7.13E-33	9.05E-31	238	32	5.4	2.26E-27	2.28E-25
KRT19	9515	2345	4	2	2165	455	3	1.58E-160	2.33E-157	2064	427	3.5	1.00E-155	1.89E-152
KRT4	14	0	14	52	23	2	8	2.19E-04	1.95E-03	22	2	7.9	3.49E-04	2.64E-03
KRT6A	492	96	5	3	849	172	4	1.74E-66	4.66E-64	750	162	3.3	4.57E-55	1.23E-52
KRT7	11141	2297	5	2	2845	465	4	1.86E-267	6.84E-264	2650	431	4.4	3.33E-250	1.88E-246
LAMB1	105	540	-5	1	37	127	-5	2.17E-20	1.57E-18	29	111	-5.3	1.94E-19	1.30E-17
LAMC1	222	971	-4	2	62	186	-4	4.12E-26	3.67E-24	56	174	-4.3	2.84E-25	2.51E-23
LAMC2	146	608	-4	1	40	127	-4	3.34E-19	2.21E-17	34	115	-4.7	2.09E-18	1.29E-16
LMNA	339	48	7	2	115	10	8	2.35E-17	1.30E-15	98	9	7.9	1.18E-14	5.30E-13
LOX	12	171	-14	-8	2	36	-25	6.41E-12	2.20E-10	2	32	-22.1	1.66E-10	4.78E-09
LRPAP1	35	0	35	1	60	3	14	2.12E-11	6.87E-10	56	1	40.5	1.46E-12	5.26E-11
MME	0	27	-27	-11	0	40	nd	8.05E-16	3.96E-14	0	38	nd	4.58E-15	2.13E-13
NDUFA1	427	14	31	1	141	5	20	3.52E-27	3.34E-25	124	3	29.9	1.32E-25	1.18E-23
NDUFA13	948	157	6	1	264	26	7	6.48E-36	8.99E-34	239	25	6.9	1.06E-31	1.36E-29
NDUFA2	275	21	13	1	70	4	13	1.13E-12	4.21E-11	64	4	11.6	2.11E-11	6.74E-10
NDUFA5	13	0	13	1	21	1	15	1.08E-04	1.04E-03	18	0	nd	5.61E-05	5.39E-04
NDUFB3	23	0	23	2	88	2	32	1.20E-18	7.44E-17	37	1	26.7	3.02E-08	6.14E-07
NDUFB8	1001	123	8	2	246	23	8	2.13E-34	2.90E-32	228	21	7.8	3.30E-32	4.29E-30
NDUFS7	334	14	24	1	72	2	26	4.89E-15	2.25E-13	65	2	23.5	1.81E-13	7.17E-12
NDUFS8	211	41	5	1	89	13	5	1.39E-10	4.06E-09	70	11	4.6	3.19E-08	6.46E-07
PDS5B	0	26	-26	1	1	34	-47	3.07E-12	1.09E-10	0	29	nd	1.14E-11	3.80E-10
PFKP	749	123	6	4	190	30	5	7.26E-20	4.97E-18	167	24	5.0	7.84E-19	5.10E-17
PHB	492	75	7	3	133	16	6	5.21E-17	2.79E-15	123	15	5.9	1.02E-15	5.02E-14
PHB2	4166	827	5	3	989	156	5	1.08E-96	4.80E-94	895	146	4.4	2.43E-85	1.20E-82
PTMA	46	0	46	1	99	2	36	3.80E-21	2.82E-19	92	2	33.2	1.48E-19	1.00E-17
PTMS	427	14	31	1	145	6	17	4.88E-27	4.57E-25	122	4	22.0	4.98E-24	4.03E-22
RAN	351	21	17	2	103	7	11	3.78E-17	2.06E-15	94	7	9.7	2.76E-15	1.31E-13
RPL10A	2949	595	5	1	910	116	6	1.27E-103	6.01E-101	849	112	5.5	1.15E-94	5.90E-92
RPL29	1229	533	2	2	906	496	1	2.83E-07	5.07E-06	816	446	1.3	9.23E-07	1.41E-05
RPL31	152	14	11	2	76	8	7	5.47E-11	1.69E-09	49	5	7.1	1.27E-07	2.30E-06
RPL38	1006	96	10	1	442	32	10	1.97E-67	5.56E-65	384	20	13.9	2.03E-65	7.65E-63
RPL39	1375	321	4	2	333	98	2	4.02E-17	2.17E-15	269	67	2.9	1.01E-17	5.85E-16
RPL41	1042	21	50	2	768	72	8	6.57E-104	3.22E-101	667	50	9.6	1.30E-99	7.34E-97
RPL8	1691	48	35	3	488	9	39	9.86E-101	4.53E-98	463	5	66.9	9.86E-101	6.20E-98
RPLP1	2475	41	60	2	912	31	21	6.97E-170	1.14E-166	859	22	28.2	2.06E-168	4.66E-165
RPS13	860	103	8	1	319	78	3	3.40E-21	2.54E-19	296	59	3.6	1.17E-24	9.77E-23
RPS15A	714	68	11	1	435	34	9	1.88E-64	4.70E-62	377	27	10.1	5.62E-58	1.67E-55
RPS19	6267	1306	5	1	1630	282	4	1.50E-146	1.30E-143	1462	220	4.8	2.05E-147	2.57E-144
RPS24	363	62	6	2	135	18	5	3.01E-16	1.51E-14	119	12	7.2	8.57E-17	4.54E-15
RPS27A	35	0	35	1	337	7	35	6.16E-69	1.78E-66	320	7	33.0	4.49E-65	1.59E-62
RPS6	170	7	24	1	64	1	46	2.14E-14	9.40E-13	60	1	43.3	1.77E-13	7.04E-12
SEMA3C	25	0	25	1	1	34	-47	3.07E-12	1.09E-10	0	27	nd	6.48E-11	1.95E-09
SFN	374	7	53	1	93	2	34	8.80E-20	5.96E-18	79	1	57.1	7.52E-18	4.43E-16
SLC3A2	257	21	12	3	83	10	6	4.06E-11	1.28E-09	71	8	6.4	5.02E-10	1.37E-08
SOS2	6	301	-50	1	9	72	-11	1.45E-18	8.80E-17	6	61	-14.1	3.93E-17	2.15E-15
SOX15	15	0	15	1	26	0	nd	7.24E-07	1.21E-05	25	0	nd	1.25E-06	1.82E-05
STAT3	64	390	-6	1	18	82	-6	2.32E-16	1.18E-14	16	72	-6.2	1.93E-14	8.45E-13
TGFBR3	6	246	-41	1	7	42	-8	3.06E-10	8.66E-09	4	35	-12.1	6.29E-10	1.67E-08
TIMP1	357	7	51	2	87	3	21	2.58E-17	1.42E-15	81	2	29.3	4.60E-17	2.49E-15
TPI1	930	34	27	2	444	57	6	3.45E-51	7.04E-49	424	56	5.5	4.43E-48	1.12E-45
TUBB2C	568	41	14	2	162	12	10	1.97E-25	1.74E-23	140	10	10.1	1.88E-22	1.46E-20
TXNIP	275	2639	-10	-3	75	539	-10	3.20E-124	2.14E-121	68	462	-9.4	3.03E-104	2.45E-101
TXNRD2	146	14	10	2	39	4	7	2.72E-06	3.97E-05	29	1	21.0	1.87E-06	2.63E-05
UCRC	30	0	30	2	57	0	nd	3.46E-14	1.50E-12	54	0	nd	1.77E-13	7.04E-12
UQCRB	556	34	16	2	207	7	21	1.13E-39	1.83E-37	196	3	47.2	4.95E-42	1.02E-39
VEGFA	23	185	-8	1	9	35	-5	4.00E-07	6.95E-06	8	33	-5.7	5.21E-07	8.41E-06
