# Supplementary figures and images for: NGS-Trex: Next Generation Sequencing Transcriptome profile explorer
Source: BMC Bioinformatics. 2013 Apr 22;14(Suppl 7):S10. doi: 10.1186/1471-2105-14-S7-S10 (PMC3633008; doi:10.1186/1471-2105-14-S7-S10)

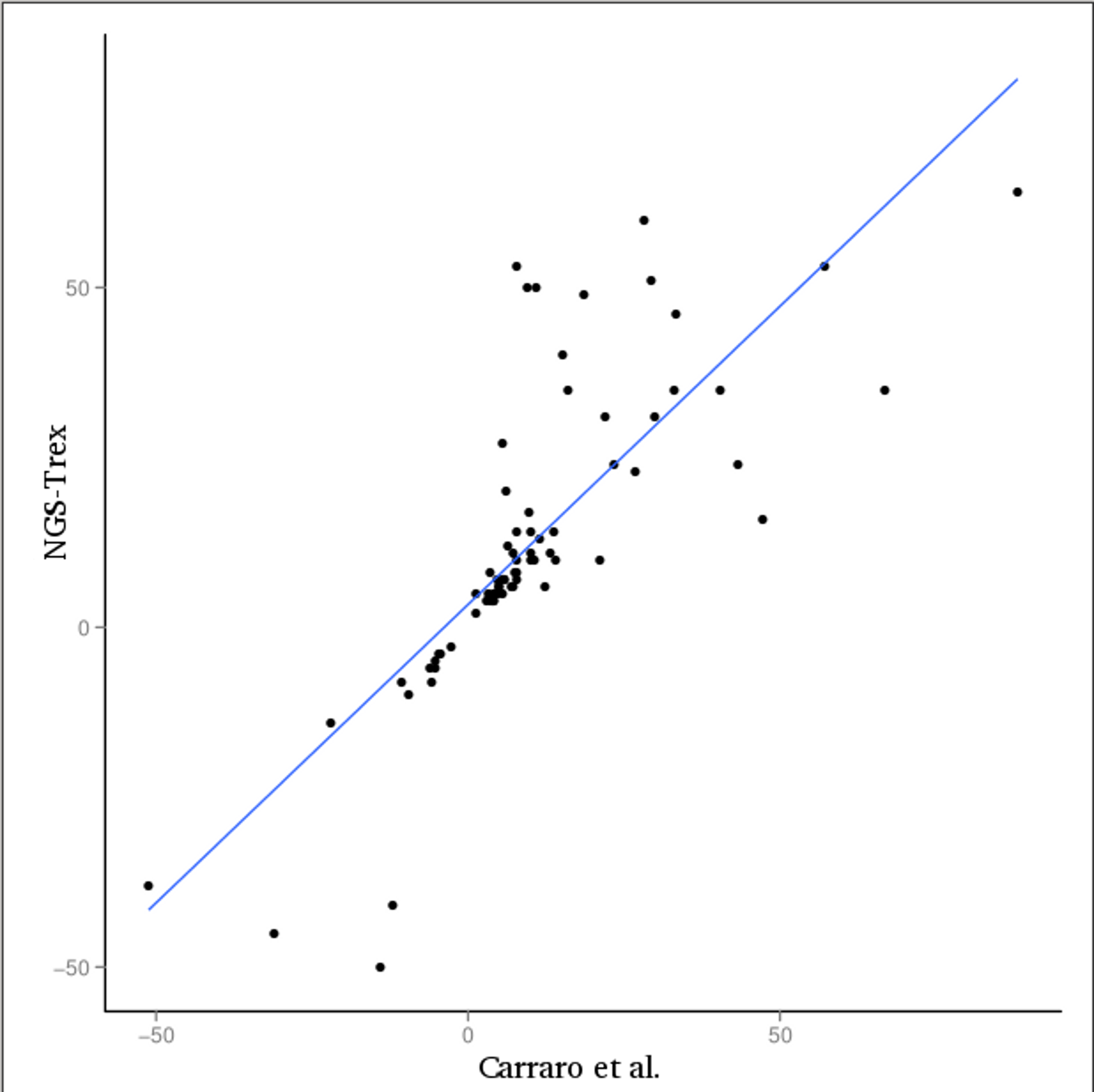

Supplement: Additional File 2 — Correlation of fold change values. The plot shows the correlation between Carraro and NGS-Trex fold changes for the 88 genes listed in Additional File 1. [file 1471-2105-14-S7-S10-S2.tiff]
